# Repurposed Antimicrobial Combination Therapy: Tobramycin-Ciprofloxacin Hybrid Augments Activity of the Anticancer Drug Mitomycin C Against Multidrug-Resistant Gram-Negative Bacteria

**DOI:** 10.3389/fmicb.2019.01556

**Published:** 2019-07-10

**Authors:** Ronald Domalaon, Derek Ammeter, Marc Brizuela, Bala Kishan Gorityala, George G. Zhanel, Frank Schweizer

**Affiliations:** ^1^Department of Chemistry, University of Manitoba, Winnipeg, MB, Canada; ^2^Department of Medical Microbiology and Infectious Diseases, University of Manitoba, Winnipeg, MB, Canada

**Keywords:** adjuvant, antibiotic hybrid, anticancer, antimicrobial, gram-negative bacteria, mitomycin C, repurposing, synergy

## Abstract

The lack of therapeutic options to treat infections caused by multidrug-resistant (MDR) pathogens, especially Gram-negative bacteria, is apparent. Therefore, it is imperative to develop new strategies to address the problem of antimicrobial resistance. Repurposing non-antibiotic commercial drugs for antimicrobial therapy presents a viable option. We screened six anticancer drugs for their potential use in antimicrobial therapy. Here, we provide *in vitro* evidence that suggests feasibility to repurpose the anticancer drug mitomycin C against MDR Gram-negative bacteria. We also demonstrated that mitomycin C, etoposide and doxorubicin were affected by drug efflux in *Pseudomonas aeruginosa*. In combination with a tobramycin-ciprofloxacin antibiotic hybrid (TOB-CIP), the antibacterial activity of mitomycin C was enhanced against MDR clinical isolates of *P. aeruginosa*, *Acinetobacter baumannii*, *Escherichia coli*, *Klebsiella pneumoniae*, and *Enterobacter cloacae*. In fact, 4 μg/mL (3 μM) TOB-CIP reduced the minimum inhibitory concentration of mitomycin C to ≤1 μg/mL against MDR Gram-negative bacteria, except *A. baumannii*. We showed that synergy was inherent to TOB-CIP and that neither tobramycin nor ciprofloxacin individually synergized with mitomycin C. Our finding supports identifying adjuvant partners for mitomycin C, such as TOB-CIP, to enhance suitability for antimicrobial therapy.

## Introduction

There is a dire need to develop new strategies that can supplement our dwindling antibiotic armamentarium to address the growing threat of antimicrobial resistance (Luepke et al., 2017; Domalaon et al., 2018a; Koulenti et al., 2018). The shortage of available options is especially dire for the treatment of MDR Gram-negative bacterial infections. In fact, the World Health Organization classified problematic Gram-negative bacteria, including carbapenem-resistant *P. aeruginosa*, carbapenem-resistant *A. baumannii* and carbapenem-resistant extended-spectrum β-lactamase-producing *Enterobacteriaceae*, to be of critical priority that urgently requires the development of antibiotics (Tacconelli et al., 2018). Promising strategies have emerged including anti-virulence therapy (Dickey et al., 2017), phage therapy (Kortright et al., 2019) and multicomponent combination therapy (Tyers and Wright, 2019). Repurposing non-antibiotic drugs for antimicrobial therapy offers a cost- and time-efficient method of discovery (Rangel-Vega et al., 2015; Soo et al., 2017; Polamreddy and Gattu, 2018; Cavalla, 2019). This is advantageous since the agents under study have well-characterized pharmacokinetic parameters and have undergone the rigorous process of safety evaluation from health agencies such as the United States’ Food and Drug Administration (FDA) (Gns et al., 2019). Several non-antibiotic drugs have been described in the literature to display potential use for the treatment of MDR Gram-negative bacterial infections (Hennessy et al., 2016; Cheng et al., 2018; Lee and O’Neill, 2018; Domalaon et al., 2019).

**FIGURE 1:**
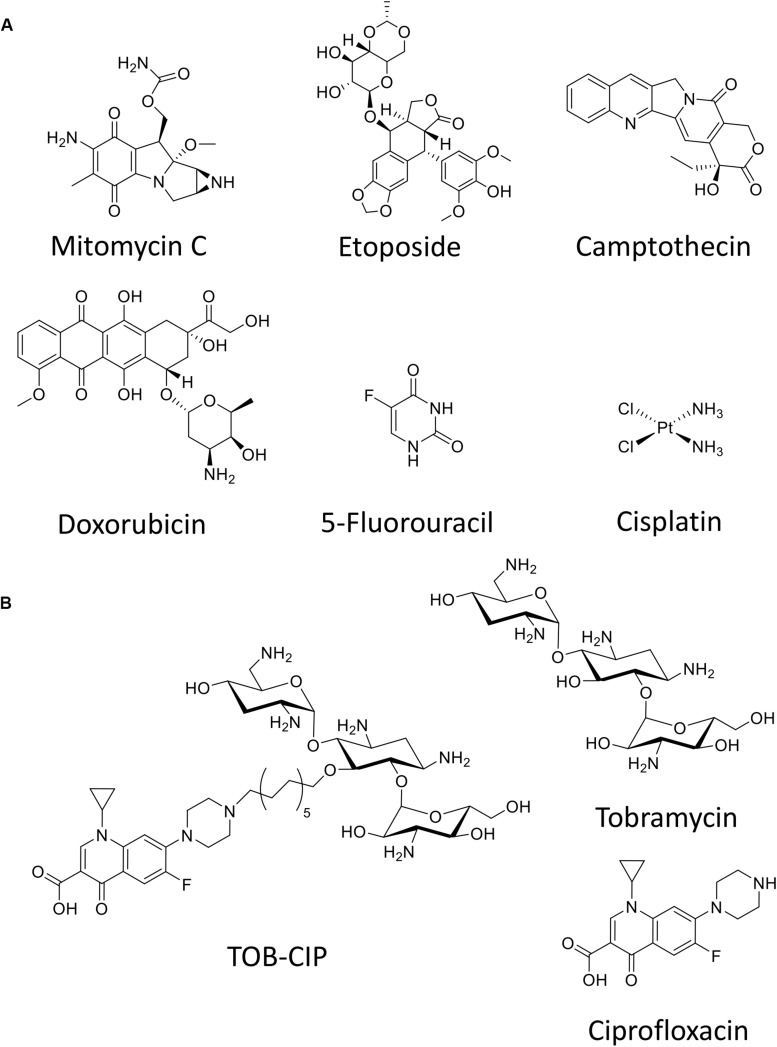
Chemical structure of **(A)** six anticancer agents evaluated in this study and **(B)** tobramycin-ciprofloxacin (TOB-CIP) hybrid along with its individual antibiotic components.

Herein, we evaluate six anticancer agents (Figure 1A) for their ability to eradicate MDR Gram-negative bacteria, as stand-alone agents and in combination with our previously reported adjuvant TOB-CIP (Figure 1B; Gorityala et al., 2016a). TOB-CIP is an antibiotic hybrid composed of the aminoglycoside tobramycin covalently attached to the fluoroquinolone ciprofloxacin via a twelve carbon-long aliphatic linker (Figure 1B). Our previous study revealed that TOB-CIP possessed weak antibacterial activity on its own but augmented the activity and efficacy of other antibiotics in combination against MDR Gram-negative bacteria, through outer membrane permeabilization, inner membrane disruption and proton motive force dissipation (Gorityala et al., 2016a). We found that the anticancer agent mitomycin C possessed potent antibacterial activity against *Pseudomonas aeruginosa*, *Escherichia coli*, *Klebsiella pneumoniae*, and *Enterobacter cloacae*, while limited activity was observed against *Acinetobacter baumannii*. Moreover, our data revealed that mitomycin C was greatly affected by efflux in *P. aeruginosa* and was a good substrate for the MexAB-OprM efflux system. More importantly, we found that TOB-CIP further enhanced the already potent antibacterial activity of mitomycin C against antibiotic susceptible and MDR clinical isolates of Gram-negative bacteria. Our findings provide evidence to consider mitomycin C for antimicrobial therapy especially against MDR Gram-negative bacteria.

## Materials and Methods

### Preparation of TOB-CIP Hybrid

The aminoglycoside tobramycin was covalently linked to the fluoroquinolone ciprofloxacin through an aliphatic hydrocarbon linker of twelve carbons-long, following our previous report (Gorityala et al., 2016a). The TOB-CIP (MW: 1131.44 g/mol for × 5 HCl salt) was characterized using one- and two-dimensional nuclear magnetic resonance and high-resolution matrix-assisted laser desorption ionization mass spectrometry experiments. Purity was then assessed for TOB-CIP using high-performance liquid chromatography and was found to be >95%.

### Bacterial Strains and Growth Conditions

Anticancer agents (mitomycin C, etoposide, camptothecin, 5-fluorouracil, cisplatin and doxorubicin) in this study were obtained from commercial sources. Bacterial strains in this study were obtained from either the American type culture collection (ATCC) or the Canadian ward surveillance (CANWARD) study (Hoban and Zhanel, 2013). Bacterial isolates belonging to the CANWARD study were isolated from clinical specimens collected from patients suffering a presumed infectious disease that were admitted in participating medical centers across Canada. Efflux-deficient *P. aeruginosa* PAO200 lacked the MexAB-OprM efflux pump while PAO750 lacked five clinically relevant pumps (MexAB-OprM, MexCD-OprJ, MexEF-OprN, MexJK, and MexXY) and the outer membrane protein OpmH. Prior to conducting microbiological experiments, bacterial isolates were grown overnight in lysogeny/luria broth (LB) on an incubator shaker at 37°C.

### Antimicrobial Susceptibility Assay

Antibacterial activity of the tested anticancer agents *in vitro* were assessed by microdilution broth susceptibility testing according to the Clinical and Laboratory Standards Institute (CLSI) guidelines (The Clinical and Laboratory Standards Institute, 2016). Bacterial cultures grown overnight were diluted in saline (0.85% NaCl) to achieve a 0.5 McFarland turbidity. Subsequently, the diluted bacterial culture was further diluted 1:50 in Mueller-Hinton broth (MHB) for inoculation to a final concentration of approximately 5 × 10^5^ colony forming units/mL. Experiments were performed on 96-well plates, in which the tested agents were 2-fold serially diluted in MHB and incubated with equal volumes of bacterial inoculum at 37°C for 18 h. Minimum inhibitory concentration (MIC) values for the tested agents were determined as the lowest concentration to inhibit visible bacterial growth in the form of turbidity, which was confirmed via EMax Plus microplate reader (Molecular Devices, United States) at 590 nm wavelength. Wells with or without bacterial cells were used as positive or negative controls, respectively.

### Checkerboard Assay

Experiments were performed on 96-well plates following previously described protocols (Domalaon et al., 2017, 2018b). The anticancer agents of interest were 2-fold serially diluted along the *x*-axis, while the TOB-CIP or other comparators were 2-fold serially diluted along the *y*-axis to create a matrix where each well consisted of a combination of both agents at different concentrations. Bacterial cultures grown overnight were then diluted in saline (0.85% NaCl) to 0.5 McFarland turbidity, subsequently followed by 1:50 further dilution in MHB and inoculation on each well to achieve a final concentration of approximately 5 × 10^5^ colony forming units/mL. Wells consisting of MHB with or without bacterial cells were used as positive or negative controls, respectively. The 96-well plates were then incubated at 37°C for 18 h and were subsequently examined for visible turbidity, which was confirmed using an EMax Plus microplate reader (Molecular Devices, United States) at 590 nm wavelength. Fractional inhibitory concentration (FIC) of the anticancer agents were calculated by dividing the MIC of anticancer agents in the presence of TOB-CIP/comparators by the MIC of anticancer agents alone. Similarly, FIC of TOB-CIP/comparators were calculated by dividing the MIC of TOB-CIP/comparators in the presence of anticancer agents by the MIC of TOB-CIP/comparators alone. The FIC index was the summation of both FIC values. An FIC index of ≤0.5, 0.5 < × < 4, or ≥4 was interpreted as synergistic, additive, or antagonistic, respectively (Odds, 2003; Kalan and Wright, 2011).

## Results

### Mitomycin C Possessed Potent Antibacterial Activity Against Gram-Negative Bacteria

The antibacterial activity of six anticancer agents were assessed against antibiotic susceptible strains (ASS) of *P. aeruginosa*, *A. baumannii*, and *E. coli* using microdilution broth assay and activities were reported as their MIC or the minimum concentration of the agents to prevent visible bacterial growth. Anticancer agents (Figure 1A) included mitomycin C, etoposide, camptothecin, 5-fluorouracil, cisplatin and doxorubicin. Antibacterial activity of the individual anticancer agents were poor overall against the three Gram-negative bacteria with the exception of mitomycin C (Table 1). The MIC of mitomycin C against *P. aeruginosa* PAO1 (2 μg/mL) and *E. coli* ATCC 25922 (0.5 μg/mL) were significantly lower than against *A. baumannii* ATCC 17978 (16 μg/mL). Nonetheless, mitomycin C displayed better activity than the other five anticancer agents.

**TABLE 1:** Minimum inhibitory concentration (MIC) of six anticancer drugs against ASS of *P. aeruginosa*, *A. baumannii*, and *E. coli*.

	**MIC, μg/mL**		
	
**Anticancer drug**	***P. aeruginosa* PAO1**	***A. baumannii* ATCC 17978**	***E. coli* ATCC 25922**
Mitomycin C	2	16	0.25
Etoposide	512	512	512
Camptothecin	256	256	256
5-Fluorouracil	64	256	32
Cisplatin	128	512	256
Doxorubicin	256	256	256

### Efflux in *P. aeruginosa* Affected the Antibacterial Activity of Mitomycin C, Etoposide and Doxorubicin

Two efflux-deficient *P. aeruginosa* strains (PAO200 and PAO750) isogenic to the ASS PAO1 were used to explore the effects of efflux on the antibacterial activity of the six anticancer agents. Strain PAO200 lacked the MexAB-OprM efflux pump while strain PAO750 lacked five clinically relevant pumps (MexAB-OprM, MexCD-OprJ, MexEF-OprN, MexJK, and MexXY) and outer membrane protein OpmH. Mitomycin C appeared to be significantly affected by efflux since a 32- and 64-fold decrease in MIC were found against PAO200 and PAO750, respectively, relative to antibiotic susceptible PAO1 (Table 2). The MICs of etoposide (Table 2) were reduced by 4- and 32-fold against PAO200 and PAO750, respectively. Conversely, an 8- and 128-fold reduction on MIC (Table 2) were observed for doxorubicin against PAO200 and PAO750, respectively. No changes in the MICs were observed for camptothecin, 5-fluorouracil and cisplatin against the efflux-deficient *P. aeruginosa* strains.

**TABLE 2:** Effect of efflux on the antibacterial activity of tested anticancer drugs against isogenic *Pseudomonas aeruginosa* strains.

	**MIC, μg/mL**		
	
**Anticancer drug**	**PAO1^a^**	**PAO200^b^**	**PAO750^c^**
Mitomycin C	2	0.062	0.031
Etoposide	512	128	16
Camptothecin	256	512	256
5-Fluorouracil	64	64	64
Cisplatin	128	128	128
Doxorubicin	256	32	2
TOB-CIP	32	4	2

*^*a*^*P. aeruginosa* antibiotic susceptible strain. ^*b*^*P. aeruginosa* efflux-deficient strain lacking the MexAB-OprM pump. ^*c*^*P. aeruginosa* efflux-deficient strain lacking five clinically relevant pumps (MexAB-OprM, MexCD-OprJ, MexEF-OprN, MexJK, and MexXY) and outer membrane protein OpmH.*

### TOB-CIP Enhanced Antibacterial Activity of Mitomycin C Against ASS of Gram-Negative Bacteria

**FIGURE 2:**
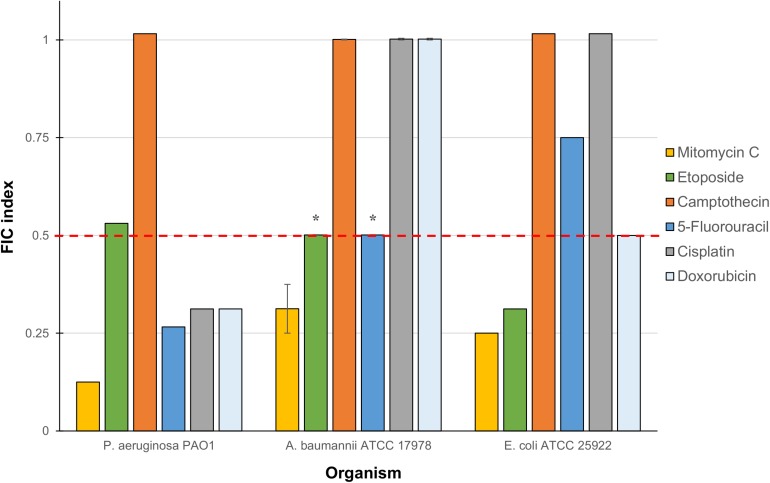
Fractional inhibitory concentration (FIC) indices of combinations consisting of TOB-CIP and either of six anticancer agents against ASS of *P. aeruginosa*, *A. baumannii*, and *E. coli*. Red dashed line denotes the cutoff FIC index of ≤0.5 for synergistic interaction. Asterisk (^*^) indicates FIC indices of 0.501 which denotes for additive interaction. See Supplementary Material for MIC and FIC values.

The six anticancer agents were then screened in combinations with our previously reported TOB-CIP hybrid (Figure 1B) for potential synergy against ASS of *P. aeruginosa*, *A. baumannii*, and *E. coli* using checkerboard assay. We wondered whether the antibacterial activity of tested anticancer agents could be further augmented, as we have previously described with combinations of clinically used antibiotics and TOB-CIP (Gorityala et al., 2016a). FIC indices for each combination were calculated; and interpreted as synergistic, additive or antagonistic for FIC indices of ≤0.5, 0.5 < × ≤ 4 and >4, respectively (Odds, 2003; Kalan and Wright, 2011). Out of the six anticancer agents, mitomycin C was consistently potentiated across the three tested ASS (Figure 2). However, it appeared that potentiation of other anticancer agents was only limited to certain organisms. Camptothecin did not synergize with TOB-CIP against antibiotic susceptible Gram-negative bacteria.

### Mitomycin C Was Not Synergized by Either Components of TOB-CIP or Other Cationic Amphiphiles

**FIGURE 3:**
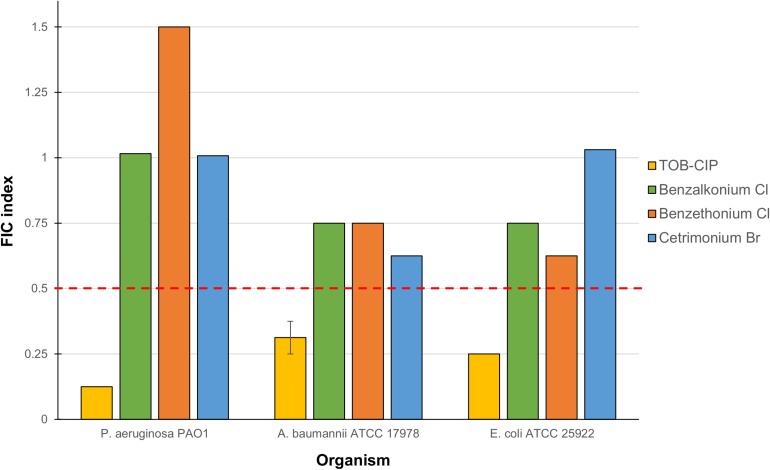
Fractional inhibitory concentration (FIC) indices of combinations consisting of mitomycin C and either TOB-CIP or commercially used amphiphiles/surfactants against ASS of *P. aeruginosa*, *A. baumannii*, and *E. coli*. Red dashed line denotes the cutoff FIC index of ≤0.5 for synergistic interaction. See Supplementary Material for MIC and FIC values.

Checkerboard assay was performed on combinations of mitomycin C and either tobramycin or ciprofloxacin against ASS of *P. aeruginosa*, *A. baumannii*, and *E. coli* to elucidate whether the observed potentiation stemmed from one or both of the TOB-CIP components (Figure 1B). Neither tobramycin nor ciprofloxacin synergized with mitomycin C against tested Gram-negative bacteria, having FIC indices ranging from 0.625 to 1.125 values (Table 3). Since our previous studies (Gorityala et al., 2016a) provided evidence that TOB-CIP potentiated antibiotics against Gram-negative bacteria by enhancing their intracellular accumulation through outer membrane permeabilization and proton-motive force disruption, we wondered whether synergy with mitomycin C could also be observed with other membrane-disputing agents. Combinations of mitomycin C and commercially used cationic amphiphiles/surfactants were then evaluated against same antibiotic susceptible Gram-negative bacteria. Cationic surfactant comparators included benzalkonium chloride, benzethonium chloride, and cetrimonium bromide. None of the comparators potentiated mitomycin C against tested ASS of *P. aeruginosa*, *A. baumannii*, and *E. coli* (Figure 3).

**TABLE 3:** Evaluation for synergy of combinations consisting of either tobramycin or ciprofloxacin and mitomycin C against ASS of *P. aeruginosa*, *A. baumannii*, and *E. coli*.

**Organism**	**MIC_Mitomycin C_ [MIC_combo_], μg/mL**	**Adjuvant**	**MIC_Adjuvant_ [MIC_combo_], μg/mL**	**FIC index**	**Interpretation**
*P. aeruginosa* PAO1	2 [1]	Tobramycin	1 [0.25]	0.750	Additive
*A. baumannii* ATCC 17978	16 [8]		1 [0.25]	0.750	Additive
*E. coli* ATCC 25922	0.25 [0.125]		2 [0.25]	0.625	Additive
*P. aeruginosa* PAO1	2 [1]	Ciprofloxacin	0.125 [0.031]	0.750	Additive
*A. baumannii* ATCC 17978	16 [8]		0.5 [0.125]	0.750	Additive
*E. coli* ATCC 25922	0.25 [0.25]		0.016 [0.002]	1.125	Additive

### TOB-CIP Potentiated Mitomycin C Against MDR Clinical Isolates of Gram-Negative Bacteria

We then assessed the combination of mitomycin C and TOB-CIP against MDR clinical isolates of Gram-negative bacteria to provide *in vitro* evidence of therapeutic utility. The panel of MDR clinical isolates included *P. aeruginosa* (5), *A. baumannii* (4)*, E. coli* (3), *K. pneumoniae* (3), and *E. cloacae* (3). Similar to what we have observed against ASS, mitomycin C displayed low MICs (Tables 4, 5) against MDR clinical isolates of *P. aeruginosa* (2–4 μg/mL) and *Enterobacteriaceae* (0.062–8 μg/mL). Conversely, the antibacterial activity of mitomycin C alone (Table 4) appeared to be limited against MDR clinical isolates of *A. baumannii* (MIC range of 8–32 μg/mL). The combination of mitomycin C and TOB-CIP was synergistic against all (5/5) *P. aeruginosa* isolates while it was synergistic against (3/4) *A. baumannii* isolates (Table 4). At a working adjuvant concentration of 4 μg/mL (3 μM) TOB-CIP, mitomycin C was potentiated up to 128-fold against *P. aeruginosa* (Table 4). It should be noted that 4 μg/mL (3 μM) was the optimal adjuvant concentration for TOB-CIP, and other tobramycin-fluoroquinolone hybrids as determined in our previous studies (Gorityala et al., 2016a,b). Against *Enterobacteriaceae*, TOB-CIP was found to synergize with mitomycin C against all (3/3) *E. coli* isolates, all (3/3) *K. pneumoniae* isolates and (2/3) *E. cloacae* isolates (Table 5). Mitomycin C potentiation at a fixed concentration of 4 μg/mL (3 μM) TOB-CIP against *Enterobacteriaceae* was found to be up to 32-fold (Table 5). Indeed, the combination of the anticancer agent mitomycin C and TOB-CIP hybrid appeared to be synergistic against antibiotic susceptible but more importantly MDR Gram-negative bacteria.

**TABLE 4:** Evaluation for synergy of combinations consisting of TOB-CIP and mitomycin C against MDR clinical isolates of *P. aeruginosa* and *A. baumannii*.

**Organism**	**MIC_Mitomycin C_ [MIC_combo_], μg/mL**	**MIC_TOB–CIP_ [MIC_combo_], μg/mL**	**FIC index**	**Interpretation**	**Absolute MIC_Mitomycin C_,^a^ μg/mL**	**Fold potentiation^b^**
*P. aeruginosa* PA259-96918	2 [0.25]	128 [8]	0.187	Synergy	0.5	4-fold
*P. aeruginosa* PA260-97103	2 [0.031]	16 [2]	0.141	Synergy	0.016	128-fold
*P. aeruginosa* PA262-101856	2 [0.25]	256 [16]	0.187	Synergy	0.5	4-fold
*P. aeruginosa* PA264-104354	2 [0.125]	64 [8]	0.187	Synergy	0.25	8-fold
*P. aeruginosa* 100036	4 [0.25]	64 [4]	0.125	Synergy	0.25	16-fold
*A. baumannii* AB027	32 [8]	>128 [2]	0.250 < × < 0.266	Synergy	8	4-fold
*A. baumannii* AB031	32 [8]	128 [16]	0.375	Synergy	16	2-fold
*A. baumannii* 92247	8 [2]	64 [32]	0.750	Additive	8	1-fold
*A. baumannii* LAC-4	8 [1]	>128 [16]	0.125 < × < 0.250	Synergy	2	4-fold

*^*a*^MIC of mitomycin C in the presence of 4 μg/mL TOB-CIP. ^*b*^Fold potentiation of mitomycin C in the presence of 4 μg/mL TOB-CIP.*

**TABLE 5:** Evaluation for synergy of combinations consisting of TOB-CIP and mitomycin C against MDR clinical isolates of *Enterobacteriaceae*.

**Organism**	**MIC_Mitomycin C_ [MIC_combo_], μg/mL**	**MIC_TOB–CIP_ [MIC_combo_], μg/mL**	**FIC index**	**Interpretation**	**Absolute MIC_Mitomycin C_,^a^ μg/mL**	**Fold potentiation^b^**
*E. coli* 94393	1 [0.125]	>128 [1]	0.125 < × < 0.133	Synergy	0.125	8-fold
*E. coli* 94474	2 [0.25]	>128 [4]	0.125 < × < 0.156	Synergy	0.25	8-fold
*E. coli* 107115	1 [0.031]	>128 [2]	0.031 < × < 0.047	Synergy	0.031	32-fold
*K. pneumoniae* 113250	1 [0.25]	>128 [16]	0.125 < × < 0.375	Synergy	0.5	2-fold
*K. pneumoniae* 113254	2 [0.25]	>128 [16]	0.125 < × < 0.250	Synergy	0.5	4-fold
*K. pneumoniae* 116381	8 [0.5]	>128 [1]	0.062 < × < 0.070	Synergy	0.25	32-fold
*E. cloacae* 117029	4 [0.5]	>128 [2]	0.125 < × < 0.156	Synergy	1	4-fold
*E. cloacae* 118564	8 [1]	>128 [1]	0.125 < × < 0.133	Synergy	1	8-fold
*E. cloacae* 121187	0.062 [0.031]	>128 [0.5]	0.500 < × < 0.504	Additive	0.031	2-fold

*^*a*^MIC of mitomycin C in the presence of 4 μg/mL TOB-CIP. ^*b*^Fold potentiation of mitomycin C in the presence of 4 μg/mL TOB-CIP.*

## Discussion

The dearth of available therapeutics to treat MDR Gram-negative bacterial infections has energized the scientific community to seek newer approaches and ingenious strategies. Instead of *de novo* development that are typically capital- and time- intensive, repurposing FDA-approved non-antibiotic drugs for antimicrobial therapy has recently gained significant traction as a method of discovery (Polamreddy and Gattu, 2018; Cavalla, 2019; Gns et al., 2019). We wondered whether anticancer agents could be repurposed as antimicrobials to eradicate MDR Gram-negative bacteria. Six anticancer agents (Figure 1A) that included DNA crosslinkers (mitomycin C and cisplatin), DNA topoisomerase blockers (etoposide, camptothecin, and doxorubicin), and thymidylate synthase inhibitors (5-fluorouracil) were evaluated against Gram-negative bacteria. Our initial assessment revealed that mitomycin C was potent against ASS of *P. aeruginosa* and *E. coli*, while moderate activity was observed against antibiotic susceptible *A. baumannii* (Table 1). The other five agents displayed poor antibacterial activity against Gram-negative bacteria (Table 1). Since anticancer agents are prone to drug efflux in rapidly dividing tumor cells (Choi and Yu, 2014; Fletcher et al., 2016), we wondered whether poor activity of these agents could be attributed to bacterial efflux systems. We probed the antibacterial activity of the six anticancer agents against efflux-deficient *P. aeruginosa* strains isogenic to antibiotic susceptible PAO1. We utilized PAO200 that lacked the MexAB-OprM efflux pump and PAO750 that lacked five clinically relevant pumps (MexAB-OprM, MexCD-OprJ, MexEF-OprN, MexJK, and MexXY) and outer membrane protein OpmH. Interestingly, the potent antibacterial activity of mitomycin C was further enhanced against the efflux pump-deletion mutants. For instance, the MIC of mitomycin C was reduced from 2 μg/mL against PAO1 to 0.062 μg/mL (32-fold reduction) against PAO200 and to 0.031 μg/mL (64-fold reduction) against PAO750 (Table 2). This significant reduction of MIC against efflux-deficient strains relative to antibiotic susceptible strain suggested that mitomycin C was a good substrate for at least the MexAB-OprM efflux system in *P. aeruginosa*. Interestingly, we found that etoposide and doxorubicin were also affected by efflux. The MICs of etoposide and doxorubicin were reduced by 4- and 8-fold, respectively, against PAO200 relative to PAO1 (Table 2). However, the MICs of etoposide and doxorubicin were more significantly reduced by 32- and 128-fold, respectively, against PAO750 relative to PAO1 (Table 2). These trends (of MIC reduction from PAO1 to PAO200 and PAO750) suggestively implied that both etoposide and doxorubicin were substrates for the MexAB-OprM efflux pump but as well as other efflux systems that were knocked-out in PAO750. Camptothecin, 5-fluorouracil and cisplatin appeared to be unaffected by bacterial efflux in *P. aeruginosa*.

We have previously reported antibiotic hybrids that could serve as adjuvants adjunct to antimicrobial therapy against Gram-negative bacteria (Domalaon et al., 2017, 2018a; Lyu et al., 2017a,b; Yang et al., 2017). Irrespective of their limited intrinsic antibacterial activity, these antibiotic hybrids efficiently permeabilize the outer membrane and perturb the inner membrane thereby resulting in dissipation of proton motive force (and de-energizing efflux systems) (Gorityala et al., 2016a, 2016b; Yang et al., 2017). Therefore, they may be used in synergistic combinations with other agents that otherwise suffer from impeded intracellular accumulation due to their limited permeation of outer membrane and/or expulsion through efflux systems. We then explored the possibility of our antibiotic hybrid TOB-CIP, composed of the aminoglycoside tobramycin covalently linked to the fluoroquinolone ciprofloxacin via twelve carbons-long aliphatic tether (Figure 1B), to enhance antibacterial activity of the six tested anticancer agents, in a drug cocktail, against Gram-negative bacteria. Checkerboard assays revealed that mitomycin C synergized with TOB-CIP against ASS of *P. aeruginosa*, *A. baumannii*, and *E. coli* (Figure 2) while synergism with the other tested agents was only limited to certain organisms. These findings are promising since mitomycin C already possessed potent antibacterial activity (low MICs) as a stand-alone agent and that the addition of TOB-CIP further lowered the needed concentration to kill Gram-negative bacteria. This prompted our study to focus on the synergistic combination of mitomycin C and TOB-CIP against MDR bacteria.

Since TOB-CIP was fundamentally composed of tobramycin and ciprofloxacin fragments (Figure 1B), we questioned whether its synergism with mitomycin C stemmed from either or both components. Our data revealed that neither tobramycin nor ciprofloxacin potentiated mitomycin C (Table 3), confirming that hybridization of the two fragments is necessary to achieve synergy. We then explored other combinations consisting of mitomycin C and other cationic amphiphiles/surfactants that were known to disrupt bacterial membranes, that included benzalkonium chloride, benzethonium chloride and cetrimonium bromide. We found that the cationic amphiphile comparators did not synergize with mitomycin C (Figure 3), suggesting that membrane disruption may not be the sole function required to yield synergy. Comparison of TOB-CIP with polymyxin B nonapeptide (PMBN), perceived to be the “gold standard” for adjuvants that act through membrane permeabilization, showed comparable results (see Supplementary Material). Both TOB-CIP and PMBN reduced the MIC of mitomycin C against *P. aeruginosa*, *A. baumannii*, and *E. coli* to similar degree at the same adjuvant concentrations. Based on our observations that mitomycin C was affected by bacterial efflux, we then questioned whether the observed synergy may be due to TOB-CIP’s ability to dissipate the proton motive force that may result in de-energized efflux systems. Note that PMBN and other polymyxins are also known to affect inner membrane components, such as vital respiratory enzyme type II NADH-quinone oxidoreductase, thereby affecting the proton motive force (Deris et al., 2014a,b). In line with this hypothesis, synergy between mitomycin C and TOB-CIP should be annulled in efflux pump-knocked out strains. Indeed, we found that the combination only yielded additive effects against efflux-deficient *P. aeruginosa* PAO200 and PAO750 strains (see Supplementary Material). Therefore, it is very likely that synergy is heavily due to TOB-CIP’s disruptive effects on efflux systems thereby enhancing intracellular accumulation of mitomycin C. However, we do not dispute that TOB-CIP’s outer membrane perturbation may also play a role. We also do not dispute that TOB-CIP may compete as a substrate for efflux (as it is also affected by efflux shown in Table 2) relative to mitomycin C, rendering the latter agent to accumulate more efficiently in the bacterial cell. In fact, we suggest that these potential mechanisms of synergy work in synchrony, with the efflux-blocking effects being a major contributor. The effect of TOB-CIP on efflux is supported by the reduction of flagellum-dependent swimming motility of *P. aeruginosa* PAO1 as flagellar function requires an intact proton motive force (Supplementary Material).

To provide preliminary evidence for therapeutic utility using *in vitro* studies, we evaluated the combination of mitomycin C and TOB-CIP against MDR clinical isolates of Gram-negative bacteria (Tables 4, 5 and Supplementary Material). The combination was found to be synergistic in all tested isolates of *P. aeruginosa* (5), *E. coli* (3), and *K. pneumoniae* (3). Mitomycin C synergized with TOB-CIP in almost all tested *A. baumannii* (4/5) and *E. cloacae* (2/3) clinical isolates. At a working adjuvant concentration of 4 μg/mL TOB-CIP, we found that the MICs of mitomycin C against *P. aeruginosa*, *E. coli*, *K. pneumoniae*, and *E. cloacae* were significantly reduced to ≤ 1 μg/mL (Tables 4, 5). In fact, the MIC of mitomycin C was potentiated 128-fold (from 2 to 0.016 μg/mL) against *P. aeruginosa* PA260-97103 in the presence of only 4 μg/mL (3 μM) TOB-CIP (Table 4). These findings suggested that the combination of mitomycin C and TOB-CIP may be of use to eradicate MDR *P. aeruginosa* and *Enterobacteriaceae*. However, the antibacterial effect of the combination appeared limited against clinical isolates of *A. baumannii* since the working adjuvant concentration of 4 μg/mL (3 μM) TOB-CIP did not lower the MIC of mitomycin C to 1 μg/mL (Table 4). Increasing the TOB-CIP concentration above 4 μg/mL may further reduce the MIC of mitomycin C, however, undesired toxicity of the adjuvant may be pronounced, as observed in our previous studies (Gorityala et al., 2016a,b).

First isolated from *Streptomyces caespitosus* in 1958 (Crooke and Bradner, 1976), mitomycin C has been an indispensable agent for cancer treatment since its FDA approval in 1974 (Bradner, 2001). It had been hinted early on (1961) that the DNA alkylating properties of the naturally occurring mitomycin C may not only be limited to fast-growing tumor cells but also to bacterial cells (Reich et al., 1960). Yet perhaps the availability of safer antibiotic alternatives, such as of the β-lactams, had relegated the therapeutic potential of mitomycin C in the past. With the increasing prevalence of MDR pathogens and the decline in available options to treat them, a revitalized interest in the antibacterial activity of mitomycin C is apparent. Our findings herein, along with others, suggests the potential of mitomycin C to be repurposed for antimicrobial therapy as either monotherapy or in combination with the adjuvant TOB-CIP. Mitomycin C have been shown to eradicate bacterial persister cells of *P. aeruginosa*, *E. coli*, and *Staphylococcus aureus* (Kwan et al., 2015). Furthermore, mitomycin C monotherapy showed efficacy in a *Caenorhabditis elegans* model of *E. coli* O157:H7 infection (Kwan et al., 2015). Another report (Cruz-Muniz et al., 2017) described the killing of MDR *A. baumannii* strains as well as the anti-biofilm properties of mitomycin C against *A. baumannii*. Moreover, it was reported that mitomycin C rescues *G. mellonella* from *A. baumannii* infections (Cruz-Muniz et al., 2017). However, antimicrobial therapy with mitomycin C is not without limitation. Peak plasma mitomycin C concentrations in humans were reported to vary per individual with a range of 0.4–3.2 μg/mL following intravenous administration (den Hartigh et al., 1983). Therefore, the effective concentration for mitomycin C to be used for antimicrobial therapy, especially against MDR Gram-negative bacteria, should be within or below this range. Based on our results, potentiators like TOB-CIP that significantly reduce the effective concentration of mitomycin C should gain broad interest to pursue this approach against MDR Gram-negative bacteria. Since our data suggest that mitomycin C is susceptible to bacterial efflux, one may postulate other favorable drug combinations that include efflux pump inhibitors and proton motive force uncouplers. Indeed, our future work includes evaluation of other adjuvants that may potentially be used in a cocktail with mitomycin C to treat complicated bacterial infections. But also, the biochemical and molecular interplay that result in synergy between mitomycin C and TOB-CIP will be elucidated.

## Data Availability

The raw data supporting the conclusions of this manuscript will be made available by the authors, without undue reservation, to any qualified researcher.

## Author Contributions

RD, GZ, and FS conceived the study. DA and BG prepared and characterized the TOB-CIP. RD and MB performed all the microbiological experiments. GZ and FS guided the progress of the study. RD wrote the manuscript with inputs from DA, MB, GZ, and FS.

## Conflict of Interest Statement

The authors declare that the research was conducted in the absence of any commercial or financial relationships that could be construed as a potential conflict of interest.
