# Supplementary material for: Repurposed Antimicrobial Combination Therapy: Tobramycin-Ciprofloxacin Hybrid Augments Activity of the Anticancer Drug Mitomycin C Against Multidrug-Resistant Gram-Negative Bacteria
Source: Front Microbiol. 2019 Jul 10;10:1556. doi: 10.3389/fmicb.2019.01556 (PMC6636613; doi:10.3389/fmicb.2019.01556)
Supplement: Supplementary file 1 [file Data_Sheet_1.PDF]

Repurposed antimicrobial combination therapy: tobramycin-ciprofloxacin hybrid augments activity of the anticancer drug mitomycin C against multidrug-resistant Gram-negative bacteria

Ronald Domalaon[1], Derek Ammeter[1], Marc Brizuela[1], Bala Kishan Gorityala[1], George G. Zhanel[2], Frank Schweizer[1,2*]

[1]Department of Chemistry, University of Manitoba, Winnipeg, MB, Canada

[2]Department of Medical Microbiology and Infectious Diseases, University of Manitoba, Winnipeg, MB, Canada

* Correspondence
Professor Frank Schweizer
schweize@cc.umanitoba.ca

Supplementary Table 1 Evaluation for synergy of combinations consisting of TOB-CIP and anticancer drugs against antibiotic susceptible strains of *P. aeruginosa*, *A. baumannii* and *E. coli*.

Drug	Organism	MIC_{Drug} [MIC_{combo}], µg/mL	$MIC_{TOB\text{-}CIP}$ [MIC_{combo}], µg/mL	FIC index	Interpretation
Mitomycin C	*P. aeruginosa* PAO1	2 [0.125]	32 [2]	0.125	Synergy
	A. baumannii ATCC 17978	16 [4]	>128 [16]	0.250<x<0.375	Synergy
	E. coli ATCC 25922	0.5 [0.062]	8 [1]	0.250	Synergy
Etoposide	*P. aeruginosa* PAO1	512 [256]	32 [1]	0.531	Additive
	A. baumannii ATCC 17978	512 [256]	>128 [0.25]	0.500<x<0.502	Additive
	E. coli ATCC 25922	512 [32]	8 [2]	0.312	Synergy
Camptothecin	*P. aeruginosa* PAO1	256 [256]	32 [0.5]	1.016	Additive
	A. baumannii ATCC 17978	256 [256]	>128 [0.25]	1.000<x<1.002	Additive
	E. coli ATCC 25922	256 [256]	8 [0.125]	1.016	Additive
5-Fluorouracil	*P. aeruginosa* PAO1	64 [16]	32 [0.5]	0.266	Synergy
	A. baumannii ATCC 17978	256 [128]	>128 [0.25]	0.500<x<0.502	Additive
	E. coli ATCC 25922	32 [16]	8 [2]	0.750	Additive
Cisplatin	*P. aeruginosa* PAO1	128 [32]	32 [2]	0.312	Synergy
	A. baumannii ATCC 17978	512 [512]	>128 [0.5]	1.000<x<1.004	Additive
	E. coli ATCC 25922	256 [256]	8 [0.125]	1.016	Additive
Doxorubicin	*P. aeruginosa* PAO1	256 [64]	32 [2]	0.312	Synergy
	A. baumannii ATCC 17978	256 [256]	>128 [0.5]	1.000<x<1.004	Additive
	E. coli ATCC 25922	256 [64]	8 [2]	0.500	Synergy

Supplementary Table 2 Evaluation for synergy of combinations consisting of TOB-CIP and mitomycin C against antibiotic susceptible and efflux-deficient strains of *P. aeruginosa*.

Drug	Organism	MIC_{Drug} [MIC_{combo}], µg/mL	$MIC_{TOB\text{-}CIP}$ [MIC_{combo}], µg/mL	FIC index	Interpretation
Mitomycin C	*P. aeruginosa* PAO1	2 [0.125]	32 [2]	0.125	Synergy
	P. aeruginosa PAO200	0.062 [0.031]	2 [0.5]	0.750	Additive
	P. aeruginosa PAO750	0.031 [0.016]	2 [0.5]	0.750	Additive

Supplementary Table 3 Evaluation for synergy of combinations consisting of mitomycin C and either TOB-CIP or commercially-used amphihiles/surfactants against antibiotic susceptible strains of *P. aeruginosa*, *A. baumannii* and *E. coli*.

Organism	$MIC_{Mitomycin\ C}$ [MIC_{combo}], µg/mL	Amphiphile	$MIC_{Amphiphile}$ [MIC_{combo}], µg/mL	FIC index	Interpretation
P. aeruginosa PAO1	2 [0.125]	TOB-CIP	32 [2]	0.125	Synergy
	2 [2]	Benzalkonium Chloride	64 [1]	1.016	Additive
	2 [2]	Benzethonium Chloride	32 [16]	1.500	Additive
	2 [2]	Cetrimonium Bromide	256 [2]	1.008	Additive
A. baumannii ATCC 17978	16 [4]	TOB-CIP	>128 [16]	0.250<x<0.375	Synergy
	16 [4]	Benzalkonium Chloride	16 [8]	0.750	Additive
	16 [4]	Benzethonium Chloride	8 [4]	0.750	Additive
	16 [2]	Cetrimonium Bromide	64 [32]	0.625	Additive
E. coli ATCC 25922	0.5 [0.062]	TOB-CIP	8 [1]	0.250	Synergy
	0.5 [0.125]	Benzalkonium Chloride	8 [4]	0.750	Additive
	0.5 [0.25]	Benzethonium Chloride	8 [1]	0.625	Additive
	0.5 [0.5]	Cetrimonium Bromide	64 [2]	1.031	Additive

Supplementary Table 4 Evaluation for synergy of combinations consisting of mitomycin C and either TOB-CIP or polymyxin B nonapeptide (PMBN) against antibiotic susceptible strains of *P. aeruginosa*, *A. baumannii* and *E. coli*.

Organism	$MIC_{Mitomycin\ C}$ [MIC_{combo}], μg/mL	Amphiphile	$MIC_{Amphiphile}$ [MIC_{combo}], μg/mL	FIC index	Interpretation
P. aeruginosa PAO1	2 [0.125]	TOB-CIP	32 [2]	0.125	Synergy
	2 [0.062]	PMBN	128 [0.016]	0.047	Synergy
A. baumannii ATCC 17978	16 [4]	TOB-CIP	>128 [16]	0.250<x<0.375	Synergy
	16 [4]	PMBN	>256 [32]	0.250<x<0.375	Synergy
E. coli ATCC 25922	0.5 [0.062]	TOB-CIP	8 [1]	0.250	Synergy
	0.5 [0.0311]	PMBN	256 [8]	0.094	Synergy

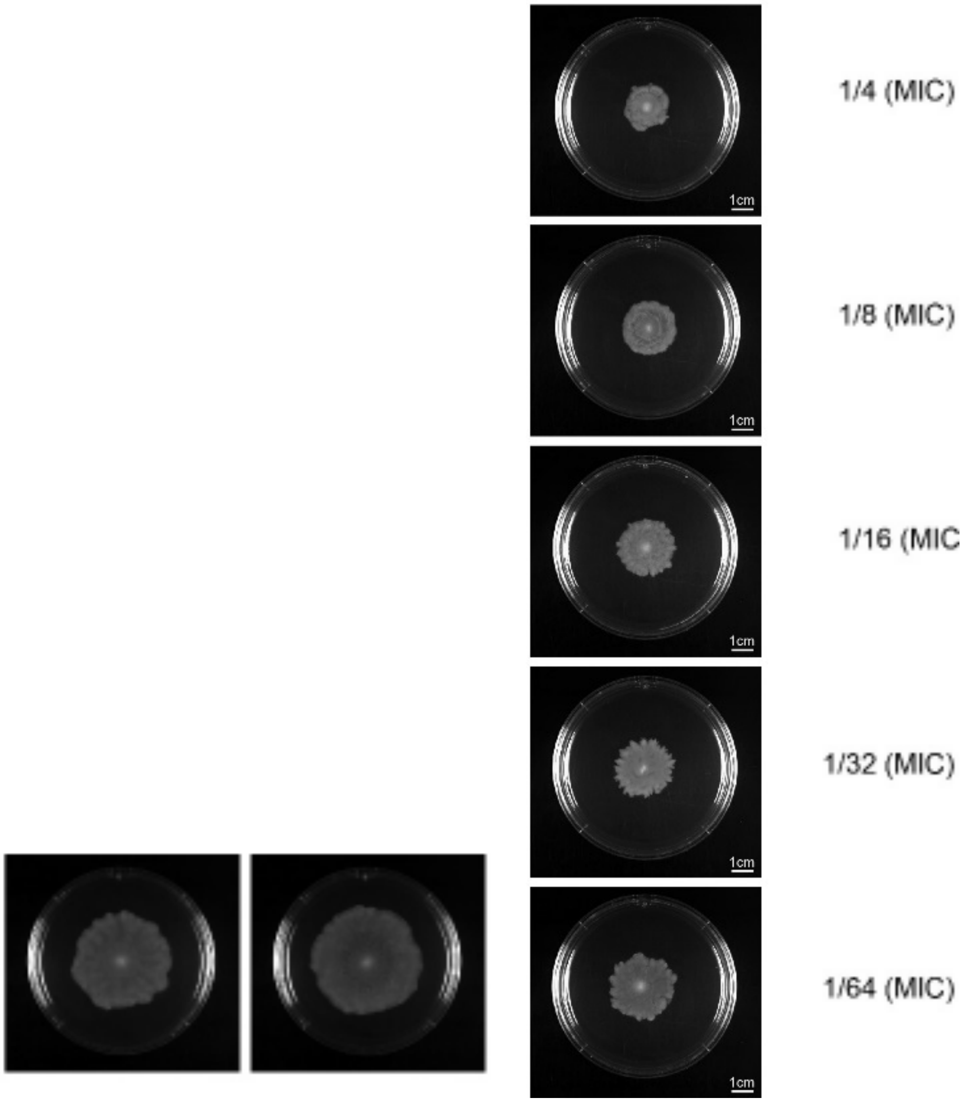

Supplementary Figure 1. Motility of *P. aeruginosa* PAO1 is greatly reduced in the presence of sub-MIC concentration of TOB-CIP. Swimming plates without conjugate serve as a control.

Swimming Motility Assay was performed as described in Yang, X et al. *J. Med. Chem. 2017,* 60, 3913−3932. In brief, the medium was composed of Trypticase Peptone (5 mg/mL), NaCl (2.5 mg/mL), and 0.3% (w/ v) agar. The motility plates were prepared by mixing the molten media with the desired concentration of the agent which was allowed to dry for an hour. 2.5 µL of an overnight grown culture of *P. aeruginosa* PAO1 was diluted in sterile PBS to an OD of 1.0. The culture was then point inoculated onto the motility plate and incubated for 20 h at 37 °C.

Supplementary Table S5 Susceptibility profiles of MDR Gram-negative bacterial clinical isolates used in the study

a) *Pseudomonas aeruginosa* isolates

Stock #	PTZ	A/C	AZT	FOX	CFZ	CTR	CPM	CAZ	IMI	MER	DOR	ETP	CIP	MOX	TOB	GEN	AMK	TGC	MIN	DOX	CST	CAM
100036	8	>32	16	>32	>128	32	4	8	8	4	16	>32	>16	>16	128	>32	32	32	16	64	2	1024
PA259-96918	64	>32	32	>32	>128	>64	>64	512	32	1024	>1024	>32	>16	>16	256	>32	>64	32	32	32	1	1024
PA260-97103	128	>32	64	>32	>128	>64	16	32	32	16	16	>32	16	>16	128	>32	4	16	16	16	1	128
PA262-101856	64	>32	32	>32	>128	64	32	16	32	32	16	>32	>16	>16	1024	>32	>64	32	64	1024	1	2048
PA264-104354	256	>32	64	>32	>128	>64	32	128	32	64	16	>32	>16	>16	128	>32	8	32	32	64	1	4096

b) *Escherichia coli* isolates

Stock #	PTZ	A/C	AZT	FOX	CFZ	CTR	CPM	CAZ	IMI	MER	ETP	CIP	MOX	TOB	GEN	AMK	TGC	DOX	CST	SXT
ATCC 25922	2	8	≤0.12	4	1	≤0.25	≤0.25	≤0.25	0.12	≤0.03	≤0.03	≤0.06	≤0.06	2	≤0.5	≤1	0.12	1	0.5	≤0.12
94393 (*mcr*-1 +ve)	≤1	4	≤0.12	4	1	≤0.25	≤0.25	≤0.25	0.25	≤0.03	≤0.03	0.5	1	≤0.5	≤0.5	2	0.25	4	4	≤0.12
94474 (*mcr*-1 +ve)	16	>32	≤0.12	16	4	≤0.25	≤0.25	0.5	0.25	≤0.03	≤0.03	>16	16	32	16	2	1	>32	16	>8
107115	>512	>32	>64	>32	>128	>64	>64	>32	8	32	>32	>16	16	8	>32	2	0.25	>32	0.5	>8

mcr = mobilized colistin resistance gene.

c) Other Gram-negative bacteria

Stock #	Organism	PTZ	A/C	AZT	FOX	CFZ	CPM	CAZ	CAZ-AVI	CTX	IMI	MER	ETP	CIP	MXF	TOB	GEN	AMK	TGC	DOX	CST	SXT
116381	*K. pneumoniae*	8	16	16	16	> 128	16	8	0.5	>64	0.5	≤0.03	0.12	>16	>16	4	≤0.5	≤1	1	>32	0.5	>8
113250	*K. pneumoniae*	4	4	≤0.12	1	1	1	0.5	ND	ND	0.25	≤0.03	≤0.03	≤0.06	≤0.06	≤0.5	≤0.5	≤1	ND	2	>16	≤0.1
113254	*K. pneumoniae*	≤1	2	≤0.12	1	1	1	≤0.2	ND	ND	0.12	≤0.03	≤0.03	≤0.06	≤0.06	≤0.5	≤0.5	≤1	ND	2	>16	≤0.1
117029	*E. cloacae*	2	16	≤0.12	>32	>128	≤0.25	0.5	0.25	≤0.25	0.25	≤ 0.03	≤0.03	≤0.06	≤0.06	2	≤0.5	2	0.5	>32	0.25	>8
118564	*E. cloacae*	2	>32	≤0.12	>32	>128	0.25	0.5	ND	ND	ND	0.12	ND	0.06	0.12	1	1	2	ND	4	>16	ND
121187	*E. cloacae*	1	8	≤0.12	>32	32	0.25	0.5	ND	ND	ND	0.06	ND	0.25	1	32	>32	1	ND	>32	>16	ND
92247	*A. baumannii*	<1	ND	ND	32	128	4	ND	ND	ND	ND	4	ND	≤0.06	ND	ND	ND	<1	0.25	ND	4	≤0.1
AB027	*A. baumannii*	512	ND	ND	ND	>128	ND	ND	ND	ND	32	16	ND	>16	8	ND	32	>64	4	ND	>256	>8
AB031	*A. baumannii*	4	ND	ND	ND	>128	ND	ND	ND	ND	0.25	1	ND	0.25	0.12	ND	<0.5	2	8	ND	16	4
LAC-4	*A. baumannii*	ND	ND	ND	ND	ND	ND	R	ND	I	S	S	ND	R	ND	R	R	I	S	S	ND	ND

PTZ: piperacillin-tazobactam; A/C: amoxicillin-clavulanic acid; AZT: aztreonam; FOX: cefoxitin; CFZ: cefazolin; CTR: ceftriaxone; CPM: cefepime; CAZ: ceftazidime; IMI: imipenem; MER: meropenem; DOR: doripenem; ETP: ertapenem; CIP: ciprofloxacin; MOX: moxifloxacin; TOB: tobramycin; GEN: gentamicin; AMK: amikacin; TGC: tigecycline; MIN: minocycline; DOX: doxycycline; CST: colistin; CAM: chloramphenicol; CTX: cefotaxime; AVI: avibactam ND: not determined; S: Susceptible; I: Intermediate; R: Resistant according to the antibiotic breakpoint guidelines of the CLSI for *Acinetobacter* spp.
